# Depression and Anxiety Among Quarantined People, Community Workers, Medical Staff, and General Population in the Early Stage of COVID-19 Epidemic

**DOI:** 10.3389/fpsyg.2021.638985

**Published:** 2021-03-26

**Authors:** Xiaoling Li, Hegao Yu, Weiqiang Yang, Qihua Mo, Zhanggui Yang, Shuangshuang Wen, Fei Zhao, Weishun Zhao, Yongyan Tang, Liang Ma, Ruifen Zeng, Xia Zou, Hanli Lin

**Affiliations:** ^1^Community Health Service Management Center, The Eighth Affiliated Hospital of Sun Yat-sen University, Shenzhen, China; ^2^Department of Otorhinolaryngology, Peking University Shenzhen Hospital, Shenzhen, China; ^3^Global Health Research Center, Guangdong Provincial People's Hospital, Guangdong Academy of Medical Sciences, Guangzhou, China

**Keywords:** depression, anxiety, COVID-19, epidemic information dissemination, risk factors

## Abstract

**Background:** We described the prevalence of anxiety and depression related to COVID-19 pandemic among different types of population and examined their potential risk factors.

**Methods:** A cross-sectional survey was conducted to collect demographic characteristics, exposure histories, and many other concerns about COVID-19. The Zung's self-rating anxiety scale (SAS) and self-rating depression scale (SDS), followed by a four-step multiple logistic regression analysis was performed to identify factors associated with mental health outcomes.

**Results:** Out of 3,303 participants, the quarantined people (40.9%), community workstation staffs-policemen-volunteers (CPV) (36.4%) and general public (30.7%) reported higher percentages of depression than the general medical staff (18.4%). Moreover, the quarantined people (19.1%) also showed higher prevalence of anxiety than the general public (9.1%) and the general medical staff (7.8%). The quarantined people had the highest risk of anxiety and depression, whereas the self-rated health was negatively associated with the risks of anxiety and depression. Younger age group (18 to 30 years) showed higher risks of anxiety (OR = 6.22, 95% CI = 2.89–13.38, *p* < 0.001) and depression (OR = 3.69, 95% CI = 2.40–5.69, *p* < 0.001). People who had exposure history or contact from Hubei province after December 1, 2019 (OR = 1.57, 95% CI = 1.07–2.30, *p* < 0.001), had family or friends engaged in front-line health care work (OR = 1.47, 95% CI = 1.02–2.14, *p* < 0.001), had confirmed case nearby (OR = 2.44, 95% CI = 1.43–4.18, *p* < 0.001) were all more likely to suffer from anxiety. Moreover, the negligence (OR = 1.85, 95% CI = 1.37–2.51, *p* < 0.001) or overindulgence (OR = 1.45, 95% CI = 1.03–2.04, *p* < 0.001) toward the epidemic information was associated with a higher risk of depression and anxiety.

**Conclusions:** Our findings show that the CPV and quarantined people were most at-risk population. We have identified that the young people, people with exposure histories and negligence or overindulgence toward epidemic information are in grave need of attention.

## Introduction

The coronavirus disease 2019 (COVID-19) emerged as a global pandemic, and by February 23, 2021, there has been over 111 million confirmed cases and 2,470,772 deaths in 223 countries around the world. By February 23, 2021, the National Health Commission of China reported 101,726 confirmed cases, with 4,842 deaths (WHO, 2021).

At the early stages of the COVID-19 epidemic, the China government rapidly implemented a series of non-medical interventional strategies to contain the disease. As an emergency containment approach, a lockdown was imposed on Wuhan on January 23, and the protocol for the community prevention and control measures for COVID-19 was released on January 25 (Nhc.gov., 2020b). The government initiated the first-level emergency response in all the 31 provinces, municipalities, and autonomous regions covering over 1.3 billion people on January 29, 2020 (Xiang et al., 2020). Facing this critical situation, all Chinese people have done their best to fight the epidemic. Many health care workers from different parts of the country were recruited and volunteered to be on the front-line and were directly engaged in the diagnosis, treatment, and care of patients with COVID-19. The community workstation, police, volunteer and community health service centers formed a Trinity Joint Prevention and Control Group to trace and quarantine all close contacts. During quarantine, the community workstation staffs, policemen, and volunteers (CPV) were responsible for managing and ensuring their daily necessities and tracks. The community health service center's medical staffs were also responsible for managing their physical and mental health. Apart from this, the general public stayed at home and socially isolated themselves to prevent spreading the infection and getting infected.

Given the high prevalence and with the rapidly increasing numbers of confirmed cases and deaths, negative emotions were spreading under this grim situation. A large number of people have been experiencing psychological problems, including anxiety, depression and stress (Kang et al., 2020; Liem et al., 2020; Xiang et al., 2020; Yang et al., 2020). Overwhelming workload, inadequate protective equipment and family concern have contributed to the mental burden of health care workers and CPV. Furthermore, this epidemic has seen entire cities in China effectively placed under lockdown with travel restrictions and mass quarantine. Separations from family and friends, the loss of freedom, and boredom have created dramatic psychological effects among citizens. Previous studies have reported a profound and wide range of psychological distress such as anxiety and depression impact on people at the individual, community, and international levels during outbreaks of SARS (Wu et al., 2009), pandemic influenza A(H1N1) (Rubin et al., 2009), and influenza A (H7N9) (Wang et al., 2014). More importantly, those people who were under quarantine because of contact with confirmed cases of SARS have reported various negative emotions during the quarantine period such as fear, sadness, anxiety and depression (Reynolds et al., 2008). Therefore, it is tempting to assume that a psychological interventional approach is urgently needed for all affected persons, including patients, health care workers, close contacts, as well as the general public.

The National Health Commission of China on January 26, 2020 issued its guideline for emergency psychological crisis intervention for people affected by COVID-19, which emphasized the need for mental health teams to deliver mental health support to patients and health care workers (Nhc.gov., 2020c). In addition, the guideline for psychological assistance hotline during the COVID-19 epidemic was released on February 7 (Nhc.gov., 2020a). In spite of imposing all these guidelines on psychological interventional strategies, studies have shown that many of the front-line health care workers involved in COVID-19 treatment and care were experiencing psychological burden, including depression, anxiety, insomnia, and distress (Lai et al., 2020). However, the exact distribution of various mental-health-problems among different groups of population is still unknown. Besides, there is no clear-cut information on the psychological impact and mental health of the persons on quarantine during the peak of the COVID-19 epidemic.

Therefore, the aim of this current study was to describe the prevalence and distribution of two major psychological problems- anxiety and depression among different groups of population, and analyzed the potential risk factors associated with these symptoms. In particular, this study will compare the differences in psychological problems between the people on quarantine and other types of population during COVID-19 epidemic. This may help government agencies and psychological experts in safeguarding the psychological well-being of people in the face of COVID-19 epidemic expansion in the world.

## Methods

### Study Design and Participants

This study is a cross-sectional survey conducted using an anonymous online questionnaire from March 5, 2020 to March 19, 2020. We employed a widely used “Sojump” platform (www.sojump.com) to generate a link to the survey questionnaire, which was distributed via WeChat (social networking software). Participants included members of the public in China with a WeChat account and aged 18 years or above. Those confirmed as COVID-19 cases, asymptomatic infections or suspected cases were excluded from the study.

The questionnaire consisted five parts, including demographic characteristics, COVID-19 epidemic-exposure histories, concerns toward the COVID-19 epidemic, the Zung's self-rating anxiety scale (SAS) and self-rating depression scale (SDS) for evaluating psychological symptoms, which took about 15 min to complete. An online written informed consent before the survey was designed to ask whether participants would like to participate. It included the aims, contents, risks and benefits of participating in this study. If they answered “yes,” the survey would begin. Otherwise, the survey was terminated. A participant was restricted to access only once for a single device.

### Ethics

This study has been approved by the Ethics Committee of the Eighth Affiliated Hospital, Sun Yat-sen University (No. 2020-001-02).

## Measurements

### Mental-Health-Problems (Outcomes)

The Chinese versions of the Zung's self-rating anxiety scale (SAS) and self-rating depression scale (SDS) were used to assess the severity of anxiety symptoms and depressive symptoms (Biggs et al., 1978; Dunstan et al., 2017; Dunstan and Scott, 2019, 2020). The SAS and the SDS covered both psychological and somatic symptoms. Participants were asked to give their responses to the questions based on their experiences and feelings during the last week. Both these scaling measures contain 20 items measured using a four-point Likert scale (1 = none, or a little of the time, 2 = some of the time, 3 = good part of the time, 4 = most, or all of the time). The raw scores range from 20 to 80 and was transformed to a standardized total score ranged from 25 to 100. A score of 50 or greater represents a reasonable cut-off point for identifying cases of anxiety and depression. Previous studies have shown that both the SAS and SDS have good internal consistency with a Cronbach's alpha of 0.83 (Dunstan and Scott, 2020) and 0.81 (Tanaka-Matsumi and Kameoka, 1986), respectively. This study has also demonstrated good internal consistency (SAS: Cronbach's alpha = 0.827; SDS: Cronbach's alpha = 0.898) and construct validity (SAS: Kaiser–Meyer–Olkin (KMO) value = 0.915; SDS: Kaiser–Meyer–Olkin (KMO) value = 0.945).

### Covariates

We collected demographic and clinical information from individuals belonging to different groups which was classified as general medical staffs, general public, front-line health care workers, CPV and quarantined people. Those directly engaged in clinical activities of diagnosing, treating, or providing nursing care to suspects with elevated temperature or confirmed cases were defined as front-line health care workers (HCWs). Demographic characteristics were collected including sex age, marital status, education level, living status and self-rated health. Self-rated health was measured using a 5-point Likert scale (1 = very good, 2 = good, 3 = general, 4 = poor, 5 = very poor). Exposure histories included: (1) exposure in Hubei province or had contacts with people from Hubei province after December 1, 2019, (2) had families or close friends engaged in front-line health care work, (3) had confirmed cases nearby. We asked participants' self-reported concern about the COVID-19 epidemic using the 5-point Likert scale ranged from 1 (not at all) to 5 (very much), and daily hours spent in attention to epidemic information.

### Statistics Analysis

Data analysis was performed using SPSS statistical software version 25.0 (IBM SPSS Statistics, New York, United States). All tests were two-tailed, with a significance level of *p* < 0.05. The categorical variables were shown as frequency and proportion (%). The Chi-square test was used to compare the demographic characteristics and different mental health statuses. A power calculation was conducted. A sample size of 3,303 can achieve 0.99 and 0.97 power to detect the difference of depression and anxiety between five groups of participants (general public, general medical staffs, front-line healthcare workers, CPV and quarantined people) using a 4 degrees of freedom Chi-Square test with a significance level of 0.05.

We conducted a four-step multiple logistic regression modeling in the following reasons. In step 1, the types of population were included in the model to evaluate the independent effects on anxiety and depression. In step 2, we added demographic variables into the model considering the difference of demographic characteristics between different types of population. In step 3, exposure history variables were added into the model considering the potential association between exposure history and mental-health-problems. In step 4, we included variables related to participants' concerns toward the epidemic into the model to explore the relationship of these variables with mental-health-problems.

## Results

### Demographic Characteristics

In total, 3,436 participants took part in the study, 3,303 participants from 34 provinces, autonomous regions and municipalities completed the survey. There have not confirmed as COVID-19 cases, asymptomatic infections and suspected cases in these participants. Of the eligible participants, 255 (7.7%) were general medical staffs, 2,413 (73.1%) general public, 204 (6.2%) front-line health care workers, 316 (9.6%) community workstation staffs-policemen-volunteers (CPV), and 115 (3.5%) quarantined people. The average age of the participants was 35.77 ± 12.45 (range 18–85). The majority of participants were female (59.2%), married (62.0%), and (64.7%) had attained a college or above educational level (Table 1).

**Table 1:** Demographic characteristics of participants.

**Variable**	**Total (*N*, %)**	**General public (*N*, %)**	**General medical staffs (*N*, %)**	**Front-line health care workers (*N*, %)**	**CPV (*N*, %)**	**Quarantined people (*N*, %)**	****χ^2^****	***P*-value**
Overall	3,303 (100.0)	2,413 (73.1)	255 (7.7)	204 (6.2)	316 (9.6)	115 (3.5)		
**Gender**							89.24	<0.001
Male	1,346 (40.8)	1,006 (41.7)	59 (23.1)	53 (26.0)	182 (57.6)	46 (40.0)		
Female	1,957 (59.2)	1,407 (58.3)	196 (76.9)	151 (74.0)	134 (42.4)	69 (60.0)		
**Age**							66.42	<0.001
18–30	1,456 (44.1)	1,089 (45.1)	96 (37.6)	75 (36.8)	150 (47.5)	46 (40.0)		
31–40	981 (29.7)	661 (27.4)	106 (41.6)	86 (42.2)	91 (28.8)	37 (32.2)		
41–50	484 (14.7)	343 (14.2)	40 (15.7)	30 (14.7)	52 (16.5)	19 (16.5)		
51–60	206 (6.2)	168 (7.0)	6 (2.4)	11 (5.4)	16 (5.1)	5 (4.3)		
>60	176 (5.3)	152 (6.3)	7 (2.7)	2 (1.0)	7 (2.2)	8 (7.0)		
**Marital status**							18.89	0.001
Single/divorced/widowed	1,255 (38.0)	948 (39.3)	70 (27.5)	64 (31.4)	130 (41.1)	43 (37.4)		
Married	2,048 (62.0)	1,465 (60.7)	185 (72.5)	140 (68.6)	186 (58.9)	72 (62.6)		
**Education**							188.50	<0.001
High school or below	1,167 (35.3)	977 (40.5)	16 (6.3)	23 (11.3)	94 (29.7)	57 (49.6)		
College or above	2,136 (64.7)	1,436 (59.5)	239 (93.7)	181 (88.7)	222 (70.3)	58 (50.4)		
**Living status**							35.24	<0.001
Alone	358 (10.8)	232 (9.6)	31 (12.2)	30 (14.7)	50 (15.8)	15 (13.0)		
Live with family member	2,733 (82.7)	2,049 (84.9)	204 (80.0)	157 (77.0)	232 (73.4)	91 (79.1)		
Live with friend	212 (6.4)	132 (5.5)	20 (7.8)	17 (8.3)	34 (10.8)	9 (7.8)		
**Self-rated health**							15.99	0.043
Excellent	2,646 (80.1)	1,923 (79.7)	207 (81.2)	162 (79.4)	262 (82.9)	92 (80.0)		
Very good	504 (15.3)	371 (15.4)	45 (17.6)	36 (17.6)	38 (12.0)	14 (12.2)		
Good/general/poor	153 (4.6)	119 (4.9)	3 (1.2)	6 (2.9)	16 (5.1)	9 (7.8)		
**Hubei exposed after December 1, 2019**							89.97	<0.001
No	3,043 (92.1)	2,270 (94.1)	232 (91.0)	159 (77.9)	289 (91.5)	93 (80.9)		
Yes	260 (7.9)	143 (5.9)	23 (9.0)	45 (22.1)	27 (8.5)	22 (19.1)		
**Family or friends engaged in front-line health care work**							202.26	<0.001
No	2,996 (90.7)	2,273 (94.2)	204 (80.0)	139 (68.1)	272 (86.1)	108 (93.9)		
Yes	307 (9.3)	140 (5.8)	51 (20.0)	65 (31.9)	44 (13.9)	7 (6.1)		
**Confirmed cases nearby**							29.83	<0.001
No	3,215 (97.3)	2,371 (98.3)	245 (96.1)	188 (92.2)	302 (95.6)	109 (94.8)		
Yes	88 (2.7)	42 (1.7)	10 (3.9)	16 (7.8)	14 (4.4)	6 (5.2)		
**Cared about the COVID-19 epidemic**							7.99	0.092
Non-care	204 (6.2)	163 (6.8)	10 (3.9)	6 (2.9)	20 (6.3)	5 (4.3)		
Care	3,099 (93.8)	2,250 (93.2)	245 (96.1)	198 (97.1)	296 (93.7)	110 (95.7)		
**Daily attention to epidemic information (hour)**							54.66	<0.001
<1	1,700 (51.5)	1,323 (54.8)	118 (46.3)	77 (37.7)	131 (41.5)	51 (44.3)		
1–3	1,157 (35.0)	802 (33.2)	102 (40.0)	81 (39.7)	132 (41.8)	40 (34.8)		
>3	446 (13.5)	288 (11.9)	35 (13.7)	46 (22.5)	53 (16.8)	24 (20.9)		
**Anxiety**^**#**^							21.66	<0.001
No	2,970 (89.9)	2,193 (90.9)	235 (92.2)	177 (86.8)	272 (86.1)	93 (80.9)		
Yes	333 (10.1)	220 (9.1)^b^	20 (7.8)^a^	27 (13.2)	44 (13.9)	22 (19.1)^ab^		
**Depression**^**#**^							29.49	<0.001
No	2,298 (69.6)	1,673 (79.3)	208 (81.6)	148 (72.5)	201 (63.6)	68 (59.1)		
Yes	1,005 (30.4)	740 (30.7)^c^	47 (18.4)^c^	56 (27.5)	115 (36.4)^c^	47 (40.9)^c^		

# Multiple comparison of anxiety and depression of five type in participants, using Bonferroni-corrected P-value (0.05/10 = 0.005).

a General medical staffs VS Quarantined people, p < 0.005;

b General public VS Quarantined people, p < 0.005;

c *General medical staffs VS General public, CPV, Quarantined people, p < 0.005*.

### Anxiety and Depression

The prevalence of anxiety (19.1%) and depression (40.9%) was the highest among quarantined people, followed by CPV (anxiety: 13.9%; depression: 36.4%), and the general public (anxiety: 9.1%; depression: 30.7%). The lowest prevalence of both anxiety and depression was among the general medical staff (anxiety: 7.8%; depression: 18.4%). The prevalence of anxiety and depression among front-line HCWs were 13.2 and 27.5%, respectively (Table 1).

### Analysis of Factors Associated With Anxiety and Depression

Quarantined people were consistently at higher risks than general medical staffs to suffer from anxiety (Model 1: OR = 2.78, 95% CI = 1.45–5.33; Model 2: OR = 2.36, 95% CI = 1.20–4.66; Model 3: OR = 2.39, 95% CI = 1.20–4.75; Model 4: OR = 2.33, 95% CI = 1.17–4.65) (Table 2) and depression (Model 1: OR = 3.06, 95% CI = 1.88–4.98; Model 2: OR = 2.49, 95% CI = 1.50–4.13; Model 3: OR = 2.57, 95% CI = 1.54–4.28; Model 4: OR = 2.52, 95% CI = 1.51–4.21) (Table 3). Besides, CPV were also at a higher risk than general medical staffs to suffer from anxiety (OR = 1.90, 95% CI = 1.09–3.32) and depression (OR = 2.13, 95% CI = 1.42–3.21), and the general public (OR = 1.70, 95% CI = 1.20–2.41) were also more likely to suffer from depression than the general medical staff.

**Table 2:** Multiple logistic regression analysis of factors associated with anxiety.

	**Model 1^**a**^**	**Model 2**	**Model 3**	**Model 4**
Pseudo *R^2^*	1.2%	8.3%	9.8%	10.7%
ΔPseudo *R^2^*	–	7.1%	1.5%	0.9%
Chi-square^b^	19.237	134.486	158.401	164.76
*P-*value	0.001	<0.001	<0.001	<0.001
**Step 1: Participants' type**				
General medical staff	Reference	Reference	Reference	Reference
General public	1.18 (0.73, 1.90)	0.97 (0.59, 1.60)	1.09 (0.66, 1.80)	1.09 (0.66, 1.80)
Front-line health care worker	1.79 (0.97, 3.30)	1.67 (0.90, 3.11)	1.41 (0.75, 2.67)	1.37 (0.72, 2.59)
CPV	1.90 (1.09, 3.32)^*^	1.50 (0.84, 2.67)	1.53 (0.86, 2.75)	1.51 (0.84, 2.70)
Quarantined people	2.78 (1.45, 5.33)^**^	2.36 (1.20, 4.66)^*^	2.39 (1.20, 4.75)^*^	2.33 (1.17, 4.65)^*^
**Step 2: Demographic**				
**Gender**				
Male	–	1.38 (1.08, 1.76)^**^	1.35 (1.06, 1.72)^*^	1.34 (1.05, 1.71)^*^
Female	–	Reference	Reference	Reference
**Age**				
18–30	–	6.78 (3.16, 14.53)^***^	6.17 (2.87, 13.24)^***^	6.22 (2.89, 13.38)^***^
31–40	–	4.62 (2.17, 9.85)^***^	4.22 (1.98, 9.00)^***^	4.27 (2.00, 9.13)^***^
41–50	–	4.16 (1.92, 9.04)^***^	3.92 (1.81, 8.50)^**^	3.93 (1.81, 8.52)^**^
51–60	–	2.89 (1.22, 6.840)^*^	2.70 (1.14, 6.40)^*^	2.66 (1.12, 6.30)^*^
>60		Reference	Reference	Reference
**Marital status**				
Single/divorced/widowed	–	Reference	Reference	Reference
Married	–	1.00 (0.74, 1.35)	1.02 (0.75, 1.38)	1.01 (0.74, 1.37)
**Education**				
High school or below	–	Reference	Reference	Reference
College or above	–	0.75 (0.58, 0.96)^*^	0.73 (0.57, 0.94)^*^	0.74 (0.57, 0.95)^*^
**Living status**				
Alone	–	Reference	Reference	Reference
Live with family member	–	0.78 (0.55, 1.11)	0.80 (0.56, 1.13)	0.81 (0.57, 1.16)
Live with friend	–	0.43 (0.23, 0.80)^**^	0.44 (0.23, 0.82)^*^	0.46 (0.24, 0.85)^*^
Self-rated health		2.50 (2.06, 3.04)^***^	2.44 (2.00, 2.96)^***^	2.43 (2.00, 2.96)^***^
**Step 3: Exposure history**				
**Hubei province exposed after December 1, 2019**				
No	–	–	Reference	Reference
Yes	–	–	1.58 (1.08, 2.32)^*^	1.57 (1.07, 2.30)^*^
**Family or close friends engaged in front-line medical work**				
No	–	–	Reference	Reference
Yes	–	–	1.51 (1.04, 2.18)^*^	1.47 (1.02, 2.14)^*^
**Confirmed cases nearby**				
No	–	–	Reference	Reference
Yes	–	–	2.42 (1.42, 4.13)^**^	2.44 (1.43, 4.18)^**^
**Step 4: Concern to the epidemic**				
**Care for the information**				
Non-care	–	–	–	1.44(0.93, 2.23)
Care	–	–	–	Reference
**Daily attention to the information**				
<1	–	–	–	Reference
1–3	–	–	–	1.12 (0.86, 1.47)
>3	–	–	–	1.45 (1.03, 2.04)^*^

a Model 1–unadjusted; Model 2–adjusted for demographics; Model 3–adjusted for demographics and epidemic-exposure variables; Model 4–adjusted for demographics, epidemic-exposure variables and epidemic-concern variables.

b Chi-square for changes in −2LnL.

* p < 0.05;

** p < 0.01;

*** *p < 0.001*.

**Table 3:** Multiple logistic regression analysis of factors associated with depression.

	**Model 1^**a**^**	**Model 2**	**Model 3**	**Model 4**
Pseudo *R^2^*	1.3%	8.0%	8.8%	10.5%
ΔPseudo *R^2^*	–	6.7%	0.8%	1.7%
Chi-square^b^	30.746	192.977	212.33	230.315
*P-*value	<0.001	<0.001	<0.001	<0.001
**Step 1: Participants' type**				
General medical staff	Reference	Reference	Reference	Reference
General public	1.96 (1.41, 2.72)^***^	1.63 (1.16, 2.29)^**^	1.74 (1.23, 2.46)^**^	1.70 (1.20, 2.41)^**^
Front-line health care worker	1.68 (1.08, 2.60)^*^	1.60 (1.02, 2.51)^*^	1.50 (0.95, 2.37)	1.50 (0.95, 2.37)
CPV	2.53 (1.71, 3.74)^***^	2.12 (1.41, 3.17)^***^	2.17 (1.44, 3.26)^***^	2.13 (1.42, 3.21)^***^
Quarantined people	3.06 (1.88, 4.98)^***^	2.49 (1.50, 4.13)^***^	2.57 (1.54, 4.28)^***^	2.52 (1.51, 4.21)^***^
**Step 2: Demographic**				
**Gender**				
Male	–	1.13 (0.97, 1.33)	1.13 (0.96, 1.32)	1.12 (0.96, 1.32)
Female	–	Reference	Reference	Reference
**Age**				
18–30	–	3.90 (2.53, 6.01)^***^	3.77 (2.44, 5.80)^***^	3.69 (2.40, 5.69)^***^
31–40	–	2.47 (1.62, 3.78)^***^	2.42 (1.58, 3.70)^***^	2.37 (1.55, 3.62)^***^
41–50	–	1.80 (1.15, 2.80)^*^	1.75 (1.12, 2.72)^*^	1.73 (1.11, 2.70)^*^
51–60	–	1.46 (0.88, 2.41)	1.42 (0.86, 2.34)	1.40 (0.84, 2.31)
>60		Reference	Reference	Reference
**Marital status**				
Single/divorced/widowed	–	Reference	Reference	Reference
Married	–	1.09 (0.89, 1.34)	1.09 (0.89, 1.34)	1.11 (0.90, 1.36)
**Education**				
High school or below	–	Reference	Reference	Reference
College or above	–	0.56 (0.50, 0.69)^***^	0.58 (0.49, 0.68)^***^	0.58 (0.49, 0.68)^***^
**Living status**				
Alone	–	Reference	Reference	Reference
Live with family member	–	0.61 (0.48, 0.78)^***^	0.62 (0.49, 0.80)^***^	0.64 (0.50, 0.82)^***^
Live with friend	–	0.47 (0.32, 0.68)^***^	0.48 (0.33, 0.70)^***^	0.51 (0.35, 0.74)^***^
Self-rated health		1.73 (1.49, 2.02)^***^	1.71 (1.47, 2.00)^***^	1.70 (1.46, 1.98)^***^
**Step 3: Exposure history**				
**Hubei province exposed after December 1, 2019**				
No	–	–	Reference	Reference
Yes	–	–	1.04 (0.78, 1.39)	1.02 (0.76, 1.37)
**Family or close friends engaged in front-line medical work**				
No	–	–	Reference	Reference
Yes	–	–	1.28 (0.98, 1.68)	1.26 (0.96, 1.66)
**Confirmed cases nearby**				
No	–	–	Reference	Reference
Yes	–	–	2.37 (1.50, 3.76)^***^	2.38 (1.50, 3.77)^***^
**Step 4: Concern to the epidemic**				
**Care the information**				
Non-care	–	–	–	1.85 (1.37, 2.51)^***^
Care	–	–	–	Reference
**Daily attention to the information**				
<1	–	–	–	Reference
1–3	–	–	–	0.96 (0.81, 1.15)
>3	–	–	–	1.17 (0.92, 1.48)

a Model 1–unadjusted; Model 2–adjusted for demographics; Model 3–adjusted for demographics and epidemic-exposure variables; Model 4–adjusted for demographics, epidemic-exposure variables and epidemic-concern variables.

b Chi-square for changes in −2LnL.

* p < 0.05;

** p < 0.01;

*** *p < 0.001*.

Self-rated health was negatively associated with the risks of anxiety (OR = 2.5, 95% CI = 2.06–3.04) and depression (OR = 1.73, 95% CI = 1.49–2.02). People at younger ages were more likely to have risks of anxiety and depression compared with people older than 60, the odds ratios of anxiety for people at 18–30, 31–40, 41–50, 51–60 years old were 6.78 (95% CI = 3.16–14.53), 4.62 (95% CI = 2.17–9.85), 4.16 (95% CI = 1.92–9.04), and 2.89 (95% CI = 1.22–6.84), respectively. Similarly, the odds ratios of depression were 3.90 (95% CI = 2.53–6.01), 2.47 (95% CI = 1.62–3.78), and 1.80 (95% CI = 1.15–2.80) for people at 18–30, 31–40, 41–50 years old than those older than 60. In addition, educational level and living status were associated with anxiety and depression.

All groups of individuals who had exposure history in Hubei province or had close contact with people from Hubei province after December 1, 2019 (OR = 1.58, 95% CI = 1.08–2.32), had family or friends engaged in front-line health care work (OR = 1.51, 95% CI = 1.04–2.18), had confirmed case nearby (OR = 2.42, 95% CI = 1.42–4.13) were more likely to suffer from anxiety compared with those groups without any of these exposure histories. Similarly, it was also noticed that the group where individual living near the confirmed cases have reported a higher risk of depression (OR = 2.37, 95% CI = 1.50–3.76) compared to all other groups.

Notably, groups with individuals who spent more time and attention on epidemic information were more likely to show anxiety symptoms (>3 vs. <1 h: OR = 1.45, 95% CI = 1.03–2.04), while those who were less concerned about epidemic information (OR = 1.85, 95% CI = 1.37–2.51) were more likely to suffer from depression.

## Discussion

The COVID-19 outbreak has disrupted the normal lives of individuals, and the worldwide rapid increase of infected cases has created a sense of uncertainty, depression, and anxiety. Mental health status of individuals engaged in front-line emergency public health events is of vital importance and utmost concern. Our study firstly reported the prevalence of anxiety and depression among general public (9.1, 30.7%), general medical staff (7.8, 18.4%), front-line health care workers (13.2, 27.5%), CPV (13.9, 36.4%), and quarantined people (19.1, 40.9%). The high prevalence of anxiety and depression was noticed among the CPV and quarantined people which may attract more concern and further actions.

The latest studies showed that during the initial stage of the COVID-19 epidemic in China (from January 29, 2020 to February 3, 2020), the prevalence of anxiety and depression of the general public and front-line health care workers were (28.8, 16.5%) and (44.6, 50.4%) (Lai et al., 2020; Wang et al., 2020). During the 2009 H1N1 pandemic more than half of health care workers reported moderately high anxiety and subsequent psychological distress (Goulia et al., 2010). Compared with these previously reported data, our study found a lower prevalence of anxiety among general public and front-line health care workers, which may be related to the improvement of diagnosis and treatment technology, the increasing availability of protective materials and the decreasing number of newly confirmed cases. Additionally, we also found that the prevalence of anxiety and depression in general medical staffs were lower than that of general public, indicating that the proper knowledge about prevention of disease can effectively reduce the occurrence of anxiety and depression.

The general public and front-line health care workers, however, are not the only ones at risk for psychological problems during this pandemic. Undoubtedly, during the lockdown and travel restrictions imposed during this epidemic outbreak, the CPV played a vital role in the prevention and control measures. Compared with the health care workers, the individuals in this group had faced more unknown sources of infections. Due to the high rate of spread of infection and the requirement of emergency critical care facilities for COVID-19, CPV were required to be on call 24 h per day to promptly investigate any suspicious person to be put under quarantine facilities. Unfortunately, the over workload, uncertainty of infection, uncooperative, and discrimination of community residents may all have extensively contributed to the mental burden of these CPV group of individuals (Ni et al., 2020).

Previous studies have identified that quarantine measures and travel restrictions can cause psychological distress and disorder such as anxiety and depression (DiGiovanni et al., 2004; Desclaux et al., 2017). Moreover, those quarantined for more than 10 days showed significantly higher psychological problems (Hawryluck et al., 2004). According to a previous study during the SARS outbreak, 28.9% of quarantined people had reported depression symptoms (Ko et al., 2006). Our finding was consistent with these study reports and highly supported that quarantine is an important factor which cause anxiety (OR = 2.33, 95% CI = 1.17–4.65) and depression (OR = 2.52, 95% CI = 1.51–4.21) among individuals. Previous studies have also showed that the upset of daily routine and reduced social and physical contact with others may often lead to boredom, frustration, and depression (Reynolds et al., 2008; Wilken et al., 2017). Moreover, progression of anxiety and depression experienced in the early stages of natural disaster can evolve into long-term post-traumatic stress disorder if not intervened at an early stage (Adams et al., 2006). Therefore, symptoms of anxiety and depression should be recognized earlier and appropriate intervention needs to be implemented immediately for the improvement of symptoms.

Of note, those individuals belonging to the group of 18–30 years of age category have reported the highest risk of anxiety and depression. This is substantiated by the fact that the psychological distress among the general population is generally found to peak around middle age (Taylor et al., 2008). The study findings would suggest that young people were particularly vulnerable and were coping less well with the consequences of this epidemic. The early stage of COVID-19 epidemic saw rapid changes in daily routines, with students moving following University closures and attending classes remotely, and for other young adults, transitioning to remote work or experiencing loss of work. These suspensions of classes, economic and employment hardships may put young people at greater risk for mental health challenges (Liu et al., 2020; Rachel Conrad et al., 2020). Besides, compared with middle-aged and elderly people, younger individuals prefer to participate in more social activities including outdoor gatherings and parties. Due to the dire need to control the spread of this epidemic, one major recommendation from health organizations was to implement social distancing procedures, which involves minimizing social and physical contact between people (WHO, 2020), making it impossible for young people participate in various social activities, which might have increased the risk of psychological problems. Young people who engaged in social distancing reported greater anxiety and depressive symptoms (Oosterhoff et al., 2020). From the general perspective of mental and physical health of younger people, it is interesting to consider the long-term consequences and potential burden of the disease.

Interestingly, we have noticed that imprudence toward the epidemic information and guide lines of prevention have increased the risk of depression, but, overindulgence and consciousness about the epidemic information for more than 3 h per day have significantly increased the risk of anxiety. As lot of misconceptions and false information regarding the epidemic outbreak are spreading around in society, most of the individuals prefer not to pay close attention to the information circulated about COVID-19. This depression might be exacerbated by the inadequate information participants often reported to be receiving from public health officials, and may confuse them about the nature of risk factors they faced (Rubin et al., 2016). However, it is also highly recommended to be over-conscious and spend a lot of time to care about the epidemic information. Moreover, too much of mixed information may lead to a difficult situation for people in finding trustworthy sources of information and may even cause harm to people's physical and mental health (Cuan-Baltazar et al., 2020). Disinformation and falsified reports about the COVID-19 have bombarded the social media and stoked unfounded fears among many netizens (Xinhuanet, 2020).

These findings implicated the government need pay more attention to mental health among CPV and quarantined people while combating with the COVID-19. In addition, it would be worthwhile to provide online or smart phone-based psycho education about the COVID-19, promote mental wellness and initiate psychological intervention (Sidi, 2020). It is noteworthy that the interventions should be implemented to help people to limit the time they spend on social media and to obtain accurate information related to the epidemic of the COVID-19 from authoritative and authentic resource to prevent psychological problems (Woon et al., 2020).

## Limitation

This study has several limitations. First, this is a cross-sectional study, so it is difficult to accurately elucidate causal relationships. It would be ideal to conduct a prospective study on the same group of participants in future to explore the possible long-term effects of quarantine. Second, because the survey was conducted online, we could not explain the questionnaires to the respondents completely, hence a possibility of respondent bias affecting the results. Finally, although the pseudo *R*^2^ of this study was very small, we have noticed that incorporating the depression or anxiety into the model have significantly increased the pseudo *R*^2^ (38.0, 30.4%). However, the focus of this study is not to explore the relationship between anxiety and depression, we hope to discover other important factors that may impact the anxiety or depression during the COVID-19 epidemic in China.

## Conclusion

Our findings show that the CPV and quarantined people were most at-risk population. We have identified that the young people, people with exposure histories and negligence or overindulgence toward epidemic information are in grave need of attention.

## Data Availability Statement

The original contributions presented in the study are included in the article/supplementary material, further inquiries can be directed to the corresponding author/s.

## Ethics Statement

The studies involving human participants were reviewed and approved by this study has been approved by the Ethics Committee of the Eighth Affiliated Hospital, Sun Yat-sen University (No. 2020-001-02). The patients/participants provided their written informed consent to participate in this study.

## Author Contributions

XL, XZ, and HL were responsible for draft writing and conceived the idea of the study. XL and XZ analyzed the data and wrote the final manuscript. XL, HL, WY, and HY were co-principal investigators designed and implemented this study. XZ and HL supervised and checked the analyses. All authors contributed to the collection of data, read, and agreed to the published version of the manuscript.

## Conflict of Interest

The authors declare that the research was conducted in the absence of any commercial or financial relationships that could be construed as a potential conflict of interest.
